# Contribution of vitamin D status as a determinant of cardiometabolic risk factors: a structural equation model, National Food and Nutrition Surveillance

**DOI:** 10.1186/s12889-021-11839-w

**Published:** 2021-10-09

**Authors:** Bahareh Nikooyeh, Tirang R. Neyestani

**Affiliations:** National Nutrition and Food Technology Research Institute and Faculty of Nutrition Science and Food Technology, Shahid Beheshti University of Medical Sciences, Tehran, 1981619573 Iran

**Keywords:** Vitamin D, Structural equation modeling, Cardiometabolic risk factors, Blood lipid profile, BMI, Surveillance

## Abstract

**Background:**

Structural equation modeling (SEM) is a method used to evaluate linear causal relationships among variables. This study aimed to investigate the direct and indirect effects of serum 25(OH) D on certain cardiovascular risk factors using SEM.

**Methods:**

An analytical cross-sectional study was conducted in six provinces of Iran. Subjects (*n* = 922), aged 19–65 years, were selected from National Food and Nutrition Surveillance. The assessments were sun-exposure behavior, anthropometric and biochemical measurements. A series of SEM models were tested and the model with the best fit indices was considered for use in the structural part of the model. Based on the literature review of previous theoretical models and supporting bivariate analyses, an overall SEM examined direct or indirect associations among observed and latent variables. We put the demographic, duration of sun exposure, anthropometric and metabolic variables in our model.

**Results:**

The paths between serum 25(OH) D and BMI were inverse and statistically significant, whereas age showed a positive association with BMI (B = 0.06, *p* < 0.001), both direct (st. effect = 0.11, *p* = 0.01) and indirect via vitamin D (st. effect = − 0.02, *p* = 0.01). The results confirmed that serum 25(OH) D concentration is a predictor for latent variable of lipid profile (B = − 0.13, *p* = 0.01) both through direct (*p* = 0.02) and indirect effects via BMI (*p* = 0.01).

**Conclusion:**

Serum 25(OH) D concentration is a predictor of BMI and also a latent variable of lipid profile via direct and indirect effects. It can also attenuate the harmful effect of age on BMI and lipid profile particularly in women.

## Background

Vitamin D is a steroid hormone and also an essential nutrient. Mother Nature has equipped human body with a machinery to build vitamin D upon skin exposure to direct sunlight. However, urbanization has broken up this natural relation between man and sun. Consequently, humans are prone to vitamin D deficiency (VDD) unless they receive sufficient amounts of the vitamin through dietary sources and supplements [1]. Among the earliest recognized functions of vitamin D, its effect on calcium homeostasis and musculoskeletal system has been well appreciated especially with the remarkable increasing rate of VDD-related osteoporosis [2, 3]. However, unlike its calcemic functions, vitamin D non-calcemic actions have been controversial [4], among which the effect on blood lipids and related cardiovascular risk has been a big argument [5–11]. Several observational studies have reported cardioprotective effects of vitamin D notably through its optimizing action on blood lipid components [12]. However, evidence for causality of this association is limited [13]. A meta-analysis reported the decreased mortality risk in those supplemented with vitamin D as compared to the controls (Relative risk = 0.93) [14]. It seems that the rise in mortality in people with low serum 25(OH) D concentrations may be particularly linked to CVD [15, 16] and its risk factors including increased blood pressure [17], blood glucose [18] and body mass index (BMI) [19]. However, reports on the relationship between circulating 25(OH) D and serum lipids as the major risk factor for CVD have been inconsistent [7, 8, 20, 21].

Considering high prevalence of suboptimal vitamin D status around the world, vitamin D supplementation as a cost-effective intervention may be a straightforwardly correctable risk factor for CVD prevention [8]. The important concern is whether vitamin D has a beneficial effect on serum lipids and cardiovascular morbidity.

The structural equation modeling (SEM) is a method used to evaluate linear causal relationships among variables and a powerful statistical tool taking into account the modeling of independent and correlated errors for examining complex research questions and analyzing the relationships among multiple variables [22]. Literally, SEM is an extension of the general linear model that enables measurement of both direct and indirect effects of variables and incorporate models with multiple dependent variables by using several regression equations simultaneously [23, 24]. This study was undertaken to investigate the direct and indirect effects of serum 25(OH) D on body mass index (BMI) and lipid profile among adult subjects using SEM approach.

## Methods

### Ethical statement

The study was conducted according to the Declaration of Helsinki. Written informed consent was obtained from all subjects prior to data collection. The protocols of National Food and Nutrition Surveillance (NFNS) were approved by the Ethics Committee of National Nutrition and Food Technology Research Institute (NNFTRI).

### Design and participants

Subjects (*n* = 922), 426 men and 496 women aged 19–65 years, were selected randomly from registered households of NFNS, a national health and nutrition program that has been implemented in Iran since 2013 by NNFTRI in collaboration with Nutrition Office of the Deputy of Health of the Ministry of Health and United Nations Children’s Fund (UNICEF). More details can be found elsewhere [25]. Briefly, participants who met the inclusion criteria (generally healthy, not using dietary supplements containing vitamin D) were randomly selected from the registered households from six provinces of Iran with different latitudes including West Azarbaijan (latitude 37.5^o^ N,45.0^o^ E), Semnan (latitude 35.5^o^ N, 53.3^o^ E), Lorestan (latitude 33.4^o^ N, 48.3^o^ E), South Khorasan (latitude 32.8^o^ N, 59.2^o^ E), Khoozestan (latitude 31.3^o^ N, 48.6^o^ E) and Fars (latitude 29.6^o^ N, 52.5^o^ E) using multistage cluster random sampling method. Data were collected in mid-winter (20 January to 20Febraury).

### Assessments

The protocols of all measurements were prepared and standardized by scientific committee of NFNS. Research teams were trained in workshop sessions held centrally to further make sure that data collection was harmonized across all regions.

### Sun exposure behavior

The questionnaire for evaluation of sun exposure behavior comprised three questions on daily sun exposure during the last 14 days including (i) whether the subject had a direct sun exposure (answers: no exposure, 10–60 min, 60–120 min, more than 120 min), (ii) if yes, at what time of day (answers: 7–10, 10–15, 15–17), and (iii) sunscreen use habits (answers: never, sometimes, often, always).

### Anthropometric measurements

Standing height was measured without shoes to the nearest of 0.1 cm. Weight was measured to the nearest of 0.1 kg and body mass index was calculated as weight (kg)/height^2^(m).

### Biochemical analyses

A venous blood sample was collected from each participant after an overnight fast. Separated serum samples were aliquoted and stored at − 80 °C until analysis day. Serum 25(OH) D concentrations were measured by enzyme immunoassay (EIA) method (Diasource, Ottignies-Louvain-la-Neuve, Belgium) with the aid a plate reader (Stat Fax 3200, Awareness, Palm City, FL, USA).

Total cholesterol (TC), high-density lipoprotein-cholesterol (HDL-C), triglycerides (TG) and low-density lipoprotein-cholesterol (LDL-C) were measured using enzymatic methods (Pars-Azmoon, Tehran, Iran) and an auto-analyzer (Selecta E; Vitalab, Holliston, Netherlands).

### Data analyses

Descriptive statistics was employed for population characteristics. Participants’ characteristics were summarized as mean ± standard deviation (SD) for continuous variables and as percentages (%) for categorical variables. Independent sample *t* test and chi-square test were used to compare variables between men and women.

### Model structure

The theoretical models that “vitamin D status influences cardio-metabolic risk factors including BMI and lipid profile” and that “sun exposure behavior and latitude of living place associate with vitamin D status” were tested using a structural equation modeling (SEM). First, we used measurement models to determine which of the serum lipid components and sun exposure questions could define the latent constructs of lipid profile and sun exposure behavior. Then, a series of SEM models were tested with direct and indirect pathways of associations between variables. The model with the best fit according to the values of several fit indices was considered for use in the structural part of the model. Gender was considered as a mediator variable. Bootstrap approaches were used to the significance of the total, direct and indirect effect among variables. The model’s goodness of fit was examined using three fit indices including relative chi-square (x^2^/df), range 2 to 5, the comparative fit index (CFI) > 0.90, and the root mean square error of approximation (RMSEA) < 0.06 [26]. Twelve subjects (1.3%) were excluded from the analysis by reason of missing data on demographics and other outcome variables. The significant differences were not detected among the selected variables (age, gender, BMI and serum 25(OH)D) between the included (*n* = 910) and excluded (*n* = 12) cases. Analyses were conducted using statistical program IBM SPSS 21.0 and IBM SPSS AMOS 20.0 (Armonk, NY). *P* value < 0.05 was considered significant for all analyses.

## Results

### Descriptive statistics

The descriptive statistics of the characteristics of the participants by gender are demonstrated in Table 1. Women had a higher BMI and serum HDL, as compared with men (*p <* 0.001). However, women exposed themselves to sun less and used sunscreen more frequently than men (*p* < 0.001). There was no significant difference in serum 25(OH) D concentrations between two genders (*p* = 0.22).

**Table 1:** The characteristics of participants based on gender

Variables	Men (*n* = 424)	Women (*n* = 486)	*p* value^*^
Age, (years)	39.3 ± 7.9	38.6 ± 8.3	0.220
Body mass index, (kg/m^2^)	26.7 ± 4.1	27.8 ± 4.8	< 0.001
Sun exposure duration, n (%)			
No exposure	45 (10.6)	115 (23.7)	< 0.001
10–60 min	164 (38.7)	279 (57.4)	
60–120 min	71 (16.7)	60 (12.3)	
More than 120 min	144 (34.0)	32 (6.6)	
Time of day (%)			
7–10	62 (14.6)	123 (25.3)	< 0.001
10–15	306 (72.2)	304 (62.6)	
15–17	56 (13.2)	59 (12.1)	
Sunscreen use (%)			
Never	383 (90.3)	199 (40.9)	< 0.001
Sometimes	25 (5.9)	120 (24.7)	
Often	4 (0.9)	55 (11.3)	
Always	12 (2.8)	112 (23.0)	
25-hydroxy vitamin D (nmol/L)	27.3 ± 15.2	25.9 ± 17.5	0.206
Triglyceride (mg/dL)	148.9 ± 83.6	114.8 ± 61.2	< 0.001
Total cholesterol (mg/dL)	169.3 ± 35.0	167.7 ± 33.1	0.485
LDL (mg/dL)	97.9 ± 28.5	95.3 ± 27.8	0.158
HDL (mg/dL)	41.7 ± 12.3	49.5 ± 11.6	< 0.001

Values are mean ± SD or number (%), *p*-values for between-group differences were assessed using t.test or chi-square test, as appropriate

### Structural equation modeling

The variables associated with the latent variable of sun exposure behavior were, in order of strength of association, duration of sun exposure, time of day and sunscreen use and latent variable of lipid profile was defined by four variables of serum TC, LDL-C, TG and HDL-C, in order of strength of association.

**Fig. 1:**
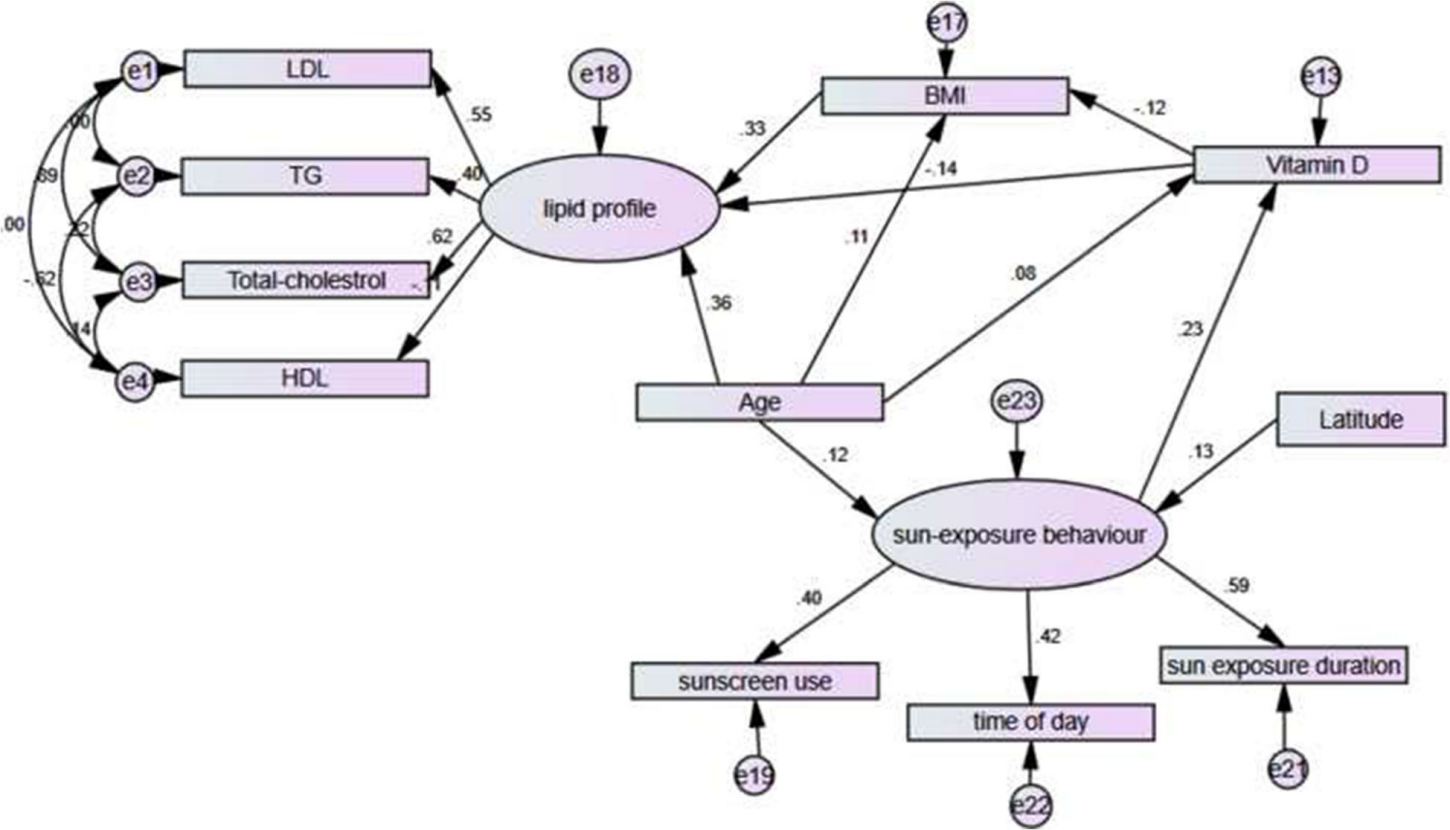
The final model with standardized parameter estimates

Based on the literature review of previous theoretical models and supporting bivariate analyses, an overall SEM examined direct or indirect associations among observed and latent variables. We put the demographic, duration of sun exposure, anthropometric and metabolic variables in our SEM (Fig. 1). The model demonstrated a good fit to the data (x^2^/df = 2.50; CFI = 0.98; TLI = 0.97; RMSEA = 0.03).

The paths between serum 25(OH) D and age (st. effect = 0.08, B = 0.15, *p* = 0.03) and sun exposure behavior (st. effect = 0.23, B = 9.73, *p* < 0.001) were statistically significant. Thus, these variables had a direct effect on vitamin D status. However, the latitude of living place did not show a significant direct effect on circulating 25(OH) D and sun exposure behavior mediated the effect of latitude on vitamin D status (st. effect = 0.03, *p* = 0.04). Moreover, 25(OH) D concentration was inversely associated with BMI, whereas age showed a positive association with BMI (B = 0.06, *p* < 0.001). The effect of age on BMI was both direct (st. effect = 0.11, *p* = 0.009) and indirect via vitamin D (st. effect = − 0.02, *p* = 0.006).

The results confirmed the hypothesis that serum 25(OH) D concentration is a predictor for latent variable of lipid profile (B = − 0.13, p = 0.006) both through direct (*p* = 0.02) and indirect effects (*p* = 0.01). The indirect effect of serum 25(OH) D on lipid profile was via BMI (Table 2).

**Table 2:** The coefficients and the corresponding *P* values of the standardized total, direct and indirect effects between variables in model

	St. total effect	*p*-value	St. direct effect	*p*-value	St. indirect effect	*p* value
Age ➞ Vit D	0.103	0.008	0.075	0.006	0.028	0.006
Age ➞ BMI	0.099	0.012	0.111	0.012	−0.012	0.005
Age ➞ lipid profile	0.377	0.006	0.358	0.009	0.019	0.251
Sun-exposure ➞ vit D	0.230	0.012	0.230	0.012	–	–
Sun-exposure ➞BMI	−0.027	0.021	–	–	−0.027	0.021
Sun-exposure ➞ lipid profile	−0.04	0.005	–	–	−0.04	0.005
Vit D ➞ BMI	−0.117	0.013	−0.117	0.013	–	–
Vit D ➞ lipid profile	−0.175	0.011	−0.136	0.023	−0.039	0.012
BMI ➞ lipid profile	0.331	0.014	0.331	0.014	–	–

### Multi-group analysis

Execution of the multi-group analysis in the model demonstrated that some paths were statistically different between men and women. Analysis revealed significant differences (*p* = 0.03) of path coefficients of serum 25(OH) D and sun exposure behaviors between men (B = 0.36, *p* = 0.01) and women (B = 0.18, *p* = 0.049). In addition, these analyses showed that age was significant predictor of BMI in women (B = 0.11, *p* < 0.001) but not in men (B = 0.01, *p* = 0.68) and path coefficients were different between two genders (*p* = 0.007). There was a significant direct effect of age on lipid profile in both men (B = 0.94, *p* < 0.001) and women (B = 0.26, *p* = 0.02), this effect was also significantly different between two genders (*p* < 0.001). However, multi-group analysis revealed no significant differences between men and women in path coefficients of vitamin D and BMI (*p* = 0.84), vitamin D and lipid profile (*p* = 0.660), or BMI and lipid profile (*p* = 0.79).

## Discussion

To our knowledge, this is the first study to provide evidence of the relationships among sun exposure behavior, serum 25(OH) D, BMI and lipid profile among adult population using SEM method. The major advantage of SEM method is its ability to examine the complex relationships among variables using a systematic approach with a simultaneous management of measurement errors [22].

### Vitamin D, sun exposure and latitude

In continuation of previous works on determinants of vitamin D status [27], we found that sun exposure behavior, independent of the latitude of living place, was an important determinant of serum 25(OH) D concentration. In addition, populations in lower latitudes tend to have lower sun exposure behavior score which may diminish their dermal vitamin D synthesis. However, multi-group analysis with the aim to compare path coefficients between men and women showed that sun exposure behavior was a stronger predictor of circulating 25(OH) D in men than in women. One unit increment in sun exposure behavior score was associated with increase in serum 25(OH) D by 45 nmol/L in men but only 8 nmol/L in women.

The effect of latitude on vitamin D status has already been reported [28, 29]. In winter, vitamin D synthesis may be insufficient and even negligible in the latitudes higher than 35 degree due to lower intensity of sunlight. Notwithstanding, a study in Korean people showed that 20–30 min of sun exposure per day during summer and fall was not adequate in achieving sufficient circulating 25(OH) D concentrations [30]. Along the same line, a study showed that sun exposure during summer when limited just to face and hands (the usual exposure sites in Iran due to cultural reasons) may not suffice to protect against vitamin D deficiency [31, 32].

### Efficiency of dermal synthesis of vitamin D according to sex

The model proposed by the current study suggests that individuals living in higher latitudes would synthesize more vitamin D in winter if they had sufficient sun exposure, as compared with the individuals who live in lower latitudes but have “sun getaway” behavior. However, circulating 25(OH) D concentrations in only a minority of the studied population reached the proposed optimal level of 50 nmol/L. In addition, our results indicate that a latent variable comprised of duration of sun exposure, time of day and sunscreen use, may adequately represent the construct of sun behavior that showed a significant association with 25(OH)D.

### Vitamin D and BMI

We found an inverse association between BMI and 25(OH) D which is in accord with some other reports [33, 34]. Previous investigations demonstrated that age, in both sexes, is an important predictor of BMI in all BMI ranges [35, 36]. However, we found that circulating 25(OH) D concentration influences the relationship between age and BMI. Along the same line, BMI has been recently proposed as an age-independent predictor of vitamin D status in women [37]. This provides an indirect linking path, in addition to the direct association.

Altogether, this observation proposes that with increasing 25(OH) D levels, the age-dependent increment in BMI may be attenuated (increasing of 10 nmol/L in serum 25(OH) D is associated with reduction of BMI by 0.3 kg/m^2^).

### Vitamin D and lipid profile

The direct and indirect effects of vitamin D on latent variable of lipid profile are noteworthy. The model showed that increasing serum 25(OH) D concentration was linked to decreased chance of lipid profile derangements. In other words, vitamin D was able to lessen the adverse effects of BMI and age on lipid profile.

Comparing our results with the existing data is not easy as most investigations have focused on association between lipid profile components and circulating 25(OH) D concentrations. The beneficial effect of vitamin D on components of blood lipid profile, CVD and myocardial infarction risk has been observed in several randomized controlled trials [38–41]. On the contrary, some studies did not report any significant effect [42–44]. As a result, the effect of vitamin D status on lipid profile has been controversial [45]. Our findings provide, for the first time through SEM approach, more convincing evidence for beneficial effects of vitamin D on blood lipids. One of the advantages of using structural equation models is the focus on latent constructs rather than on variables used to measure these constructs. Unlike previous studies, we considered the modulating effect of vitamin D on lipid profile as a latent construct rather than its components as single variables.

## Conclusion

Altogether, these observations propose that serum 25(OH) D concentration is a predictor of latent variable of lipid profile and BMI via direct and indirect effects. Also, with increasing serum 25(OH) D concentrations, the age-dependent increment in BMI and dyslipidemia may be attenuated. In addition, vitamin D may be able to lessen the adverse effects of BMI and age on lipid profile. Nevertheless, the cross-sectional nature of this study hinders a conclusive evaluation of causal associations. Though SEM is applied to evaluate linear causal relationships among variables [22], precise evaluation of causality still warrants further clinical trial studies.

## Data Availability

The datasets used and/or analyzed during the current study are available from the corresponding author on reasonable request.
